# Quantifying CD73 expression after chemotherapy or chemoradiotherapy in esophageal squamous cell carcinoma

**DOI:** 10.1007/s12672-025-02179-x

**Published:** 2025-03-29

**Authors:** Zachary A. Cooper, Ying Wang, Philip L. Martin, Kosho Murayama, Rakesh Kumar, Ken Kato, Shun Yamamoto, Shigeki Sekine

**Affiliations:** 1 Oncology R&D, AstraZeneca, 1 Medimmune Way, Gaithersburg, MD USA; 2 Oncology R&D, AstraZeneca, 35 Gatehouse Drive, Waltham, MA USA; 3 Oncology R&D, AstraZeneca K.K., 3-1 Ofuku-Cho, Kita-Ku, Osaka, 530-0011 Japan; 4 Department of Head and Neck, Esophageal Medical Oncology, National Cancer Center Hospital, 5 Chome-1-1 Tsukiji, Chuo City, Tokyo, 104-0045 Japan; 5 Department of Diagnostic Pathology, National Cancer Center Hospital, 5 Chome-1-1 Tsukiji, Chuo City, Tokyo, 104-0045 Japan

**Keywords:** Adenosine, CD73, Esophageal squamous cell carcinoma, Neoadjuvant chemotherapy, Neoadjuvant chemoradiotherapy

## Abstract

**Background:**

CD73 and CD39, key components of the adenosine axis, are expressed in multiple malignancies; the impact of standard-of-care treatment on their expression and antitumor immunity in esophageal squamous cell carcinoma (ESCC) remains unclear. We evaluated the adenosine axis in the context of neoadjuvant therapy received and its relationship to immune markers in ESCC tumor samples.

**Methods:**

Samples from patients who underwent surgical resection at the National Cancer Center Hospital, Tokyo, Japan, between January 2002 and July 2019 following no neoadjuvant therapy (n = 55; treatment-naïve), chemotherapy (n = 200), or chemoradiotherapy (CRT; n = 20) were immunohistochemically stained for CD73, CD39, PD-L1, FoxP3, and CD8; markers were quantified across tumor microenvironment (TME) compartments.

**Results:**

Median CD73 TME expression was lower in the treatment-naïve (2.8%) versus chemotherapy (7.2%; p < 0.0001) and CRT (6.4%; p < 0.01) cohorts, most profoundly in the stroma (median 4.1% vs 9.4% [p < 0.0001] and 8.1% [p < 0.01]). Median intraepithelial CD8-positive cell density was higher in the treatment-naïve (200.7 cells/mm^2^) versus chemotherapy (93.9 cells/mm^2^; p < 0.0001) and CRT (30.5 cells/mm^2^; p < 0.001) cohorts. Three-year recurrence-free survival (RFS) was 73.0%, 58.0%, and 30.0%, and 3-year overall survival (OS) was 78.2%, 71.4%, and 33.5%, in the treatment-naïve, chemotherapy, and CRT cohorts, respectively. High versus low CD73 TME expression was prognostic for longer RFS (treatment-naïve cohort: hazard ratio [HR] 0.16, 95% confidence interval [CI] 0.05–0.58, p = 0.0014; chemotherapy cohort: HR 0.52, 95% CI 0.34–0.78, p = 0.0012) and OS.

**Conclusions:**

These translational data demonstrating higher CD73 expression in tumors after neoadjuvant chemotherapy or CRT support potential combination strategies with CD73-targeted treatment in ESCC.

**Supplementary Information:**

The online version contains supplementary material available at 10.1007/s12672-025-02179-x.

## Introduction

In Western countries, treatment of resectable locally advanced esophageal squamous cell carcinoma (ESCC) generally comprises neoadjuvant chemoradiotherapy (CRT) with or without surgical resection [1]. However, risk of recurrence is high in patients who do not achieve pathologic complete response (pCR), and adjuvant nivolumab for 1 year is recommended for patients with residual pathologic disease [2]. In Japan and several other Asian countries, primary treatment for resectable locally advanced esophageal cancer involves neoadjuvant chemotherapy followed by surgery. Adjuvant nivolumab for 1 year is also approved in Japan for patients who do not achieve pCR after surgery [3]. However, even with intensive perioperative therapy, the rate of recurrence remains high, and the overall prognosis for patients with esophageal cancer remains poor. Therefore, there is a substantial unmet clinical need for new therapies.

Adenosine is an immunosuppressive autocrine and paracrine factor that accumulates in the tumor microenvironment (TME) [4, 5]. Upon apoptotic or necrotic cell death, tumor cells release adenosine triphosphate (ATP) into the extracellular space, resulting in a pro-inflammatory response [4, 6]. Cluster of differentiation (CD)39 and CD73 nucleotidases catalyze ATP to adenosine monophosphate (AMP) and AMP to immunosuppressive adenosine, respectively [7, 8]. The clinical significance of CD73 expression has been demonstrated in multiple tumor cell types [9, 10], with high tumoral expression of CD73 being an independent prognostic factor for worse disease-free survival (DFS) and overall survival (OS) in ESCC [11]. Additionally, numerous factors have been associated with the regulation and increased expression of CD73 including hypoxia, transforming growth factor beta (TGFβ), and prostaglandin E2 (PGE2) [12–14].

Radiotherapy has demonstrated increased tumoral expression of CD73 and programmed death-ligand 1 (PD-L1) [15–17] and CD73 gene expression is upregulated in radioresistant esophageal cancer cell lines [18]. Additionally, cytotoxic chemotherapy such as carboplatin, doxorubicin, gemcitabine, or paclitaxel induces a marked increase in CD47-, CD73-, and PD-L1-positive breast cancer cells in vitro via induction of both mRNA and protein levels [19]. In preclinical models, radiotherapy combined with anti-CD73 therapy, with or without anti-PD-(L)1 therapy, increases antitumor activity versus immunotherapy alone [16, 17].

However, CD73 expression after chemotherapy or CRT and its prognostic significance in ESCC are not clear. The objective of this study was to evaluate the effects of neoadjuvant chemotherapy or CRT on the expression of CD73 and other immune markers and to correlate these findings with clinicopathological parameters, recurrence-free survival (RFS), and OS.

## Materials and methods

### Patient cohorts

Tumor samples were obtained from a retrospectively identified cohort of patients with ESCC who underwent surgical resection at the National Cancer Center Hospital, Tokyo, Japan, between January 2002 and July 2019. All patients from whom samples were obtained had a confirmed diagnosis of ESCC by histology.

Cohorts of patients were defined according to therapy received prior to surgical resection: no therapy (treatment-naïve cohort), neoadjuvant chemotherapy (chemotherapy cohort), or neoadjuvant CRT (CRT cohort). Clinical data, including information on patient demographics, disease characteristics, and neoadjuvant therapy received, were retrospectively extracted from a prospectively maintained database at the National Cancer Center Hospital Japan. Patient data were verified and disease recurrence status was updated at each follow-up clinic visit, which occurred every 4–6 months. The clinical data cut-off date for RFS and OS in the current analyses was June 8, 2022.

### Tumor sample analysis: pathology and immunohistochemistry

The surgically resected tumor samples from patients with ESCC were evaluated for tumor size, differentiation, lymph node involvement, margins status, and pathologic stage. All parameters were reported by a pathologist at the National Cancer Center Hospital, Japan using the standardized pathology reporting system. Pathologic stages were grouped according to American Joint Committee on Cancer (AJCC)/Union for International Cancer Control (UICC) Tumor, Node, and Metastasis (TNM) classification, 8th edition [20].

Immunohistochemistry (IHC) analyses were done on 4 μm-thick sections cut from a hematoxylin and eosin-stained representative tumor block for each patient. PD-L1 expression was tested using the Ventana antibody clone SP263 IHC assay on a Ventana Benchmark Auto-stainer (Roche Diagnostics, Ventana Medical Systems, Tucson, AZ). CD73 and CD39 IHC were performed with rabbit monoclonal antibody clones D7F9A (Cell signaling, #13160) and EPR20461 (Abcam, #ab223843), respectively, on a Leica bond platform (Leica Biosystems, Wetzlar, Germany). CD8-purple (Dako clone M7103) and FoxP3-DAB (Abcam, #ab99963, clone SP97) IHC was performed using a three-plex chromogenic IHC assay with Pan Cytokeratin-yellow (PanCK; clone AE1/AE3/PCK26, Ventana 760–2135, Ventana Discovery Ultra) for automated quantification of immune cells in the epithelial versus the stromal compartment of the TME.

All IHC stained slides were converted into high-resolution digital images of the whole section (e-slides) using Aperio AT Turbo or Aperio XT scanners (Leica Biosystems, Buffalo Grove, IL) with a 20 × objective magnification. Digital images were manually annotated to designate the tumor regions for analysis. Marker quantification was performed by image analysis using HALO® artificial intelligence (AI) software (Indica Labs, Albuquerque, NM) employing customized algorithms for CD73, CD39, PD-L1, and CD8/FoxP3/PanCK. CD73 expression was assessed as percentage CD73-positive tissue in the annotated area for the total area, or for the epithelium, stroma, or immune compartments individually (necrotic area excluded). CD8-positive and FoxP3-positive cell densities from the CD8/FoxP3/PanCK three-plex were reported as marker-positive cells/mm^2^ of tumor region in the tumor epithelium and non-epithelium (i.e., stroma and immune compartments). PD-L1 and CD39 were assessed as percentage marker-positive tissue in the annotated area.

### Statistical analysis

RFS and OS were assessed for each patient cohort using Kaplan–Meier methodology. To assess the impact of adenosine and immune biomarkers on RFS and OS, outcomes were analyzed for patients in the treatment-naïve and chemotherapy cohorts in ‘marker-high’ and ‘marker-low’ subgroups defined by the median value of the respective biomarker. Markers evaluated in subgroup analyses were the percentages of the tumor samples comprising tumor epithelium, stroma, and immune infiltrate, the percentage CD73-positive tissue in the TME overall and in the tumor epithelium, stroma, and immune infiltrate separately, CD8-positive cell density in the tumor epithelium and stroma, FoxP3-positive cell density in the tumor epithelium and stroma, percentage PD-L1-positive tissue in the TME overall, and percentage CD39-DAB-positive tissue in the TME overall. Univariant Cox proportional hazard models were used to compute the hazard ratios (HRs) and 95% confidence intervals (CIs) for the comparisons of RFS and OS in marker-high versus marker-low subgroups. Two-tailed t-tests were used to compare means of continuous variables between two groups and one-way analyses of variances (ANOVA) were used for comparisons across multiple groups. P-values of less than 0.05 were considered statistically significant for tumor assessments. Statistical analyses were performed using GraphPad Prism or R-4.1.2 [21]. Survival analyses were run using the R “survival” package [21, 22].

## Results

### Patients

Tumor samples from a total of 275 patients with ESCC were included in this analysis; 55 patients had received no neoadjuvant therapy (treatment-naïve cohort), and 200 and 20 had received neoadjuvant chemotherapy or CRT, respectively. Patient demographics were comparable across cohorts (Table 1). Pathologic T status was 1A and 1B in 2 (3.6%) and 23 (41.8%) patients in the treatment-naïve cohort, 5 (2.5%) and 50 (25.0%) patients in the chemotherapy cohort, and no patients in the CRT cohort; pathologic N status was 0 in 35 (63.6%), 21 (10.5%), and 5 (25.0%) patients, respectively; and pathologic stage was IA and IB in 23 (41.8%) and 1 (1.8%) patients, respectively, in the treatment-naïve cohort, no patients in the chemotherapy cohort, and 3 (15.0%) and no patients in the CRT cohort. The most commonly used chemotherapy regimens across the chemotherapy and CRT cohorts were cisplatin plus 5-fluorouracil (5-FU; *n* = 173/220; 78.6%) and docetaxel plus cisplatin plus 5-FU (*n* = 46/220; 20.9%).

**Table 1:** Patient demographics and disease characteristics

Characteristic	Treatment-naïve cohort (*N* = 55)	Chemotherapy cohort (*N* = 200)	CRT cohort (*N* = 20)
Sex, n (%)			
Male	43 (78.2)	171 (85.5)	16 (80.0)
Female	12 (21.8)	29 (14.5)	4 (20.0)
Age group, n (%)			
≥ 65 years	34 (61.8)	105 (52.5)	8 (40.0)
< 65 years	21 (38.2)	95 (47.5)	12 (60.0)
Smoking status, n (%)			
Smoker	35 (63.6)	164 (82.0)	19 (95.0)
Non-smoker	20 (36.4)	36 (18.0)	1 (5.0)
Pathologic T status, n (%)			
1A	2 (3.6)	5 (2.5)	0
1B	23 (41.8)	50 (25.0)	0
2	1 (1.8)	11 (5.5)	1 (5.0)
3	27 (49.1)	134 (67.0)	19 (95.0)
4	2 (3.6)	0	0
Pathologic N status, n (%)			
0	35 (63.6)	21 (10.5)	5 (25.0)
1	11 (20.0)	118 (59.0)	10 (50.0)
2	9 (16.4)	57 (28.5)	5 (25.0)
3	0	4 (2.0)	0
Pathologic stage, n (%)			
IA	23 (41.8)	0	3 (15.0)
IB	1 (1.8)	0	0
IIA	11 (20.0)	21 (10.5)	7 (35.0)
IIB	2 (3.6)	58 (29.0)	2 (10.0)
IIIA	9 (16.4)	68 (34.0)	7 (35.0)
IIIB	7 (12.7)	49 (24.5)	0
IIIC	1 (1.8)	4 (2.0)	0
IV	1 (1.8)	0	1 (5.0)
Tumor differentiation, n (%)			
Well differentiated	16 (29.1)	43 (21.5)	3 (15.0)
Moderately differentiated	30 (54.5)	112 (56.0)	16 (80.0)
Poorly differentiated	9 (16.4)	17 (8.5)	1 (5.0)
Microinvasive	0	28 (14.0)	0
Neoadjuvant chemotherapy, n (%)			
Cisplatin + 5-FU	NA	154 (77.0)	19 (95.0)
Docetaxel + cisplatin + 5-FU	NA	45 (22.5)	1 (5.0)
Nedaplatin + 5-FU	NA	1 (0.5)	0

*5-FU* 5-Fluorouracil, *CRT* chemoradiotherapy, *NA* not applicable

### Marker expression in the TME on IHC

**Fig. 1:**
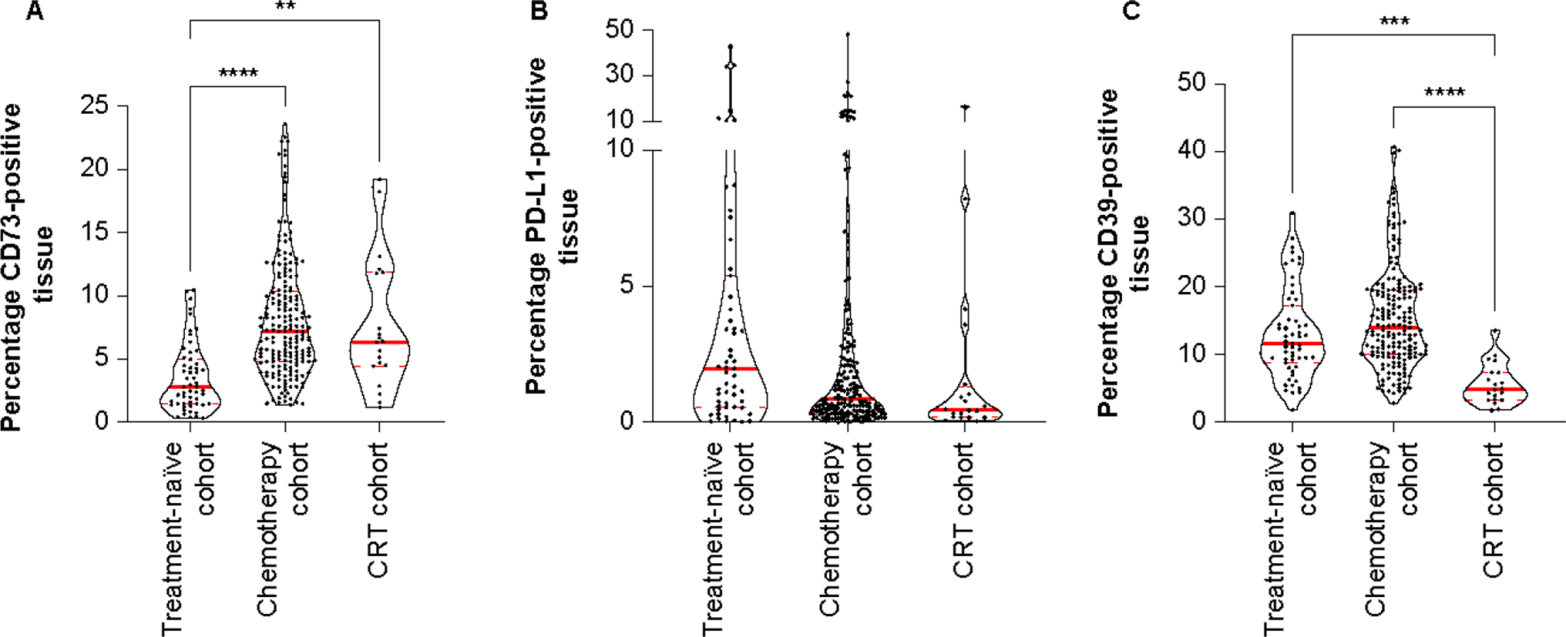
Expression of **A** CD73, **B** PD-L1, and **C** CD39 in the TME of resected ESCC tumor samples from patients who received no treatment, chemotherapy, or CRT prior to surgery. **A** CD73 expression was increased in the chemotherapy and CRT cohorts versus the treatment-naïve cohort. **B** There were no significant differences in PD-L1 expression across cohorts. **C** CD39 expression was lower in the CRT cohort versus the treatment-naïve and chemotherapy cohorts. Solid red lines represent the medians; dashed red lines represent the quartiles. *p < 0.05; **p < 0.01, ***p < 0.001, ****p < 0.0001. *CD* cluster of differentiation, *CRT* chemoradiotherapy, *ESCC* esophageal squamous cell carcinoma, *PD-L1* programmed death-ligand 1, *TME* tumor microenvironment

Image analysis of IHC stained tumor samples for CD73, CD39, PD-L1, and CD8/FoxP3/PanCK in the TME showed that median CD73 expression in the TME overall was higher in the chemotherapy (7.2% CD73-positive tissue) and CRT (6.4%) cohorts compared with the treatment-naïve cohort (2.8%; p < 0.0001 and p < 0.01, respectively) (Fig. 1A). No significant differences in PD-L1 expression were observed between cohorts (Fig. 1B). Median CD39 expression was lower in the CRT cohort (4.9% CD39-positive tissue) compared with either the chemotherapy (14.0%, p < 0.0001) or treatment-naïve (11.7%, p < 0.001) cohorts (Fig. 1C).

**Fig. 2:**
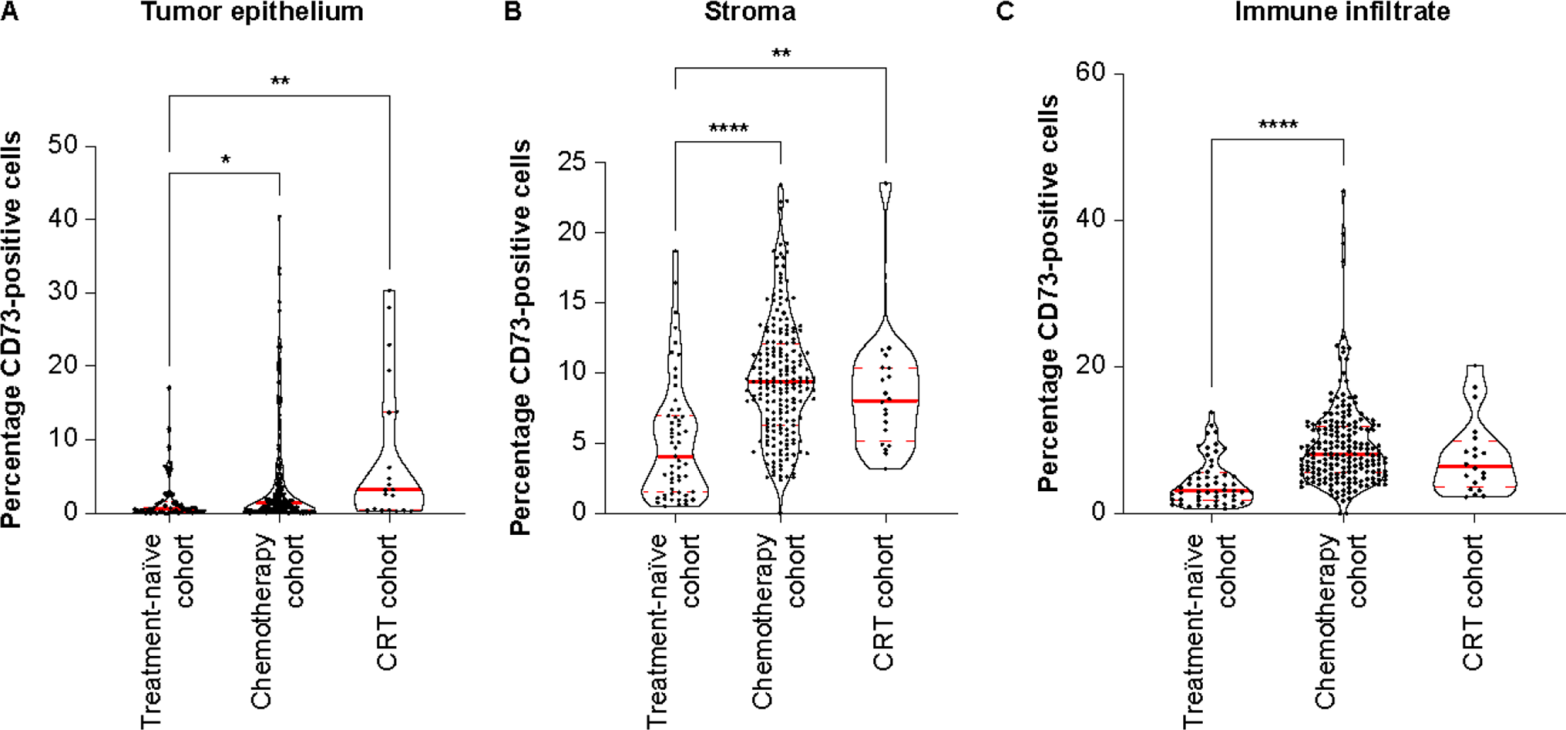
Expression of CD73 in **A** tumor epithelium, **B** stroma, and **C** immune infiltrate compartments of resected ESCC tumor samples from patients who received no treatment, chemotherapy, or CRT prior to surgery. **A**, **B** CD73 expression was increased in the tumor epithelium and stroma in the chemotherapy and CRT cohorts versus the treatment-naïve cohort. **C** CD73 expression was increased in the immune infiltrate in the chemotherapy cohort versus the treatment-naïve cohort. Solid red lines represent the medians; dashed red lines represents quartiles. *p < 0.05; **p < 0.01, ***p < 0.001, ****p < 0.0001. *CD* cluster of differentiation, *CRT* chemoradiotherapy, *ESCC* esophageal squamous cell carcinoma

Using HALO^®^ AI digital image analysis software to classify and annotate the tumor epithelium, stroma, necrotic areas, and immune infiltrates and to assess the expression of CD73 across different compartments (Supplementary Fig. 1) demonstrated that CD73 expression was primarily observed in stromal and immune cells compared with in epithelial tumor cells (Fig. 2). Tumor samples from patients in the chemotherapy and CRT cohorts showed higher CD73 expression in the stroma (median CD73-positive area: 9.3% and 8.1% vs 4.1%; p < 0.0001 and p < 0.01, respectively) (Fig. 2B) and tumor epithelium (1.5% and 3.2% vs 0.6%; p < 0.05 and p < 0.01, respectively) (Fig. 2A), and CD73 expression was also higher in the immune infiltrate for the chemotherapy versus treatment-naïve cohort (median 8.0% vs 3.2%, p < 0.0001) (Fig. 2C). The difference in CD73 expression between cohorts appeared to be most profound in the stroma.

**Fig. 3:**
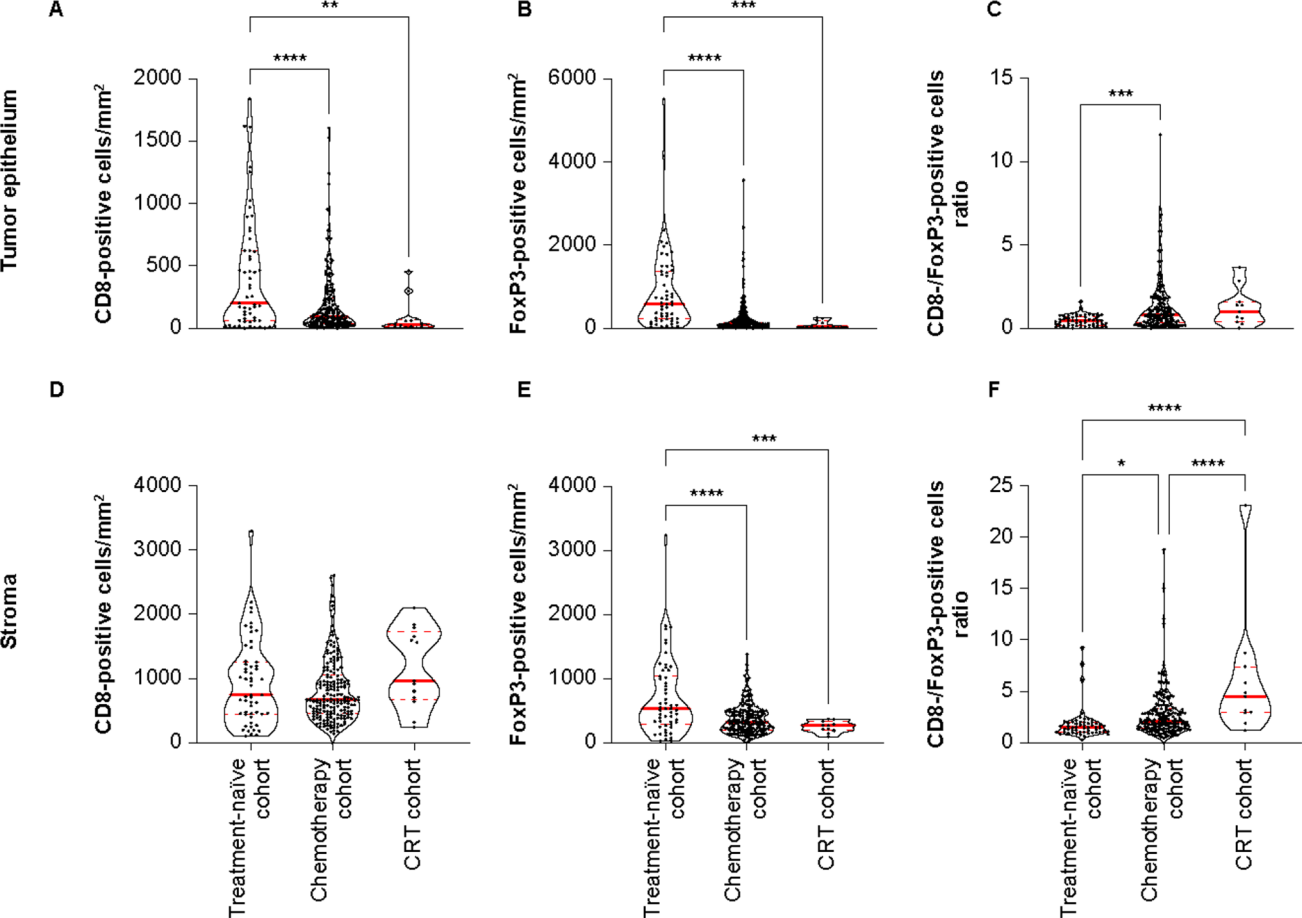
Tumor epithelium and stromal **A**, **D** density of CD8-positive cells, **B**, **D** density of FoxP3-positive cells, and **C**, **F** CD8/FoxP3 ratio in resected ESCC tumor samples from patients who received no treatment, chemotherapy, or CRT prior to surgery. **A** CD8-positive cell density was lower in the tumor epithelium in the chemotherapy and CRT cohorts versus the treatment-naïve cohort, but **D** there were no significant differences in stromal CD8-positive cell density across cohorts. **B**, **E** FoxP3-positive cell density was lower in the tumor epithelium and stroma in the chemotherapy and CRT cohorts versus the treatment-naïve cohort. The CD8/FoxP3-positive cells ratio **c** in the tumor epithelium was higher in the chemotherapy cohort versus the treatment-naïve cohort and **F** in the stroma was higher in the chemotherapy and CRT cohorts versus the treatment-naïve cohort. Solid red lines represent the medians; dashed red lines represent the quartiles. *p < 0.05; **p < 0.01, ***p < 0.001, ****p < 0.0001. *CD* cluster of differentiation, *CRT* chemoradiotherapy, *ESCC* esophageal squamous cell carcinoma

In the treatment-naïve cohort, median CD8-positive cell density was higher in the stroma (745.6 cells/mm^2^) than in the tumor epithelium (200.7 cells/mm^2^) (p < 0.0001; Supplementary Fig. 2a). In contrast, median Foxp3-positive cell densities were similar (529.8 vs 579.2 cells/mm^2^) between compartments (Supplementary Fig. 2b). CD8-positive cell density was significantly lower in the tumor epithelium in the chemotherapy and CRT cohorts compared with the treatment naïve cohort (median 93.9 and 30.5 cells/mm^2^ vs 200.7 cells/mm^2^, p < 0.0001 and p < 0.01, respectively) (Fig. 3A), whereas there were no significant differences in stromal CD8-positive cell density between the different cohorts (Fig. 3D). In contrast, FoxP3-positive cell densities were significantly lower in both the epithelial (median 118.3 and 36.6 vs 579.2 cells/mm^2^, p < 0.0001 and p < 0.001, respectively) (Fig. 3B) and stromal (median 324.3 and 267.9 vs 529.8 cells/mm^2^, p < 0.0001 and p < 0.001, respectively) (Fig. 3E) compartments in the chemotherapy and CRT cohorts compared with the treatment-naïve cohort. As CD8-positive cells were abundant in the stromal compartment compared with the epithelium, the differential effect of chemotherapy and CRT on CD8-positive cells compared with FoxP3-positive cells resulted in significantly increased CD8/FoxP3 ratios in the tumor epithelium in the chemotherapy versus treatment-naïve cohort (median ratio 0.8 vs 0.4, p < 0.001) (Fig. 3C) and in the stroma in both the chemotherapy and CRT versus tumor-naïve cohorts (2.1 and 4.5 vs 1.5, p < 0.05 and p < 0.0001, respectively) and in the CRT versus chemotherapy cohort (p < 0.0001) (Fig. 3F).

### Clinical outcomes by TME marker expression

Median RFS was not reached (NR), 95.9 months, and 6.3 months in the treatment-naïve, chemotherapy, and CRT cohorts, respectively, and 3-year RFS rates (95% CIs) were 73.0% (61.8–86.2), 58.0% (51.5–65.4), and 30.0% (15.4–58.6), respectively (Supplementary Fig. 3A, C, E). Median OS was 99.6, 141.2, and 25.6 months in the treatment-naïve, chemotherapy, and CRT cohorts, respectively, and 3-year OS rates (95% CIs) were 78.2% (67.5–90.5), 71.4% (65.2–78.3), and 33.5% (17.5–64.3), respectively (Supplementary Fig. 3b, d, f).

Analysis of RFS and OS in the treatment-naïve and chemotherapy cohorts according to marker levels, dichotomized around the median, showed that the percentage tumor epithelium in a sample was a negative biomarker and that the percentage of CD73-positive tissue in the TME was a prognostic biomarker for both RFS and OS in both cohorts. CD8-positive cell density, FoxP3-positive cell density, percentage CD39-DAB-positive tissue, and percentage PD-L1-positive tissue did not show any prognostic association in these cohorts (Supplementary Fig. 4).

**Fig. 4:**
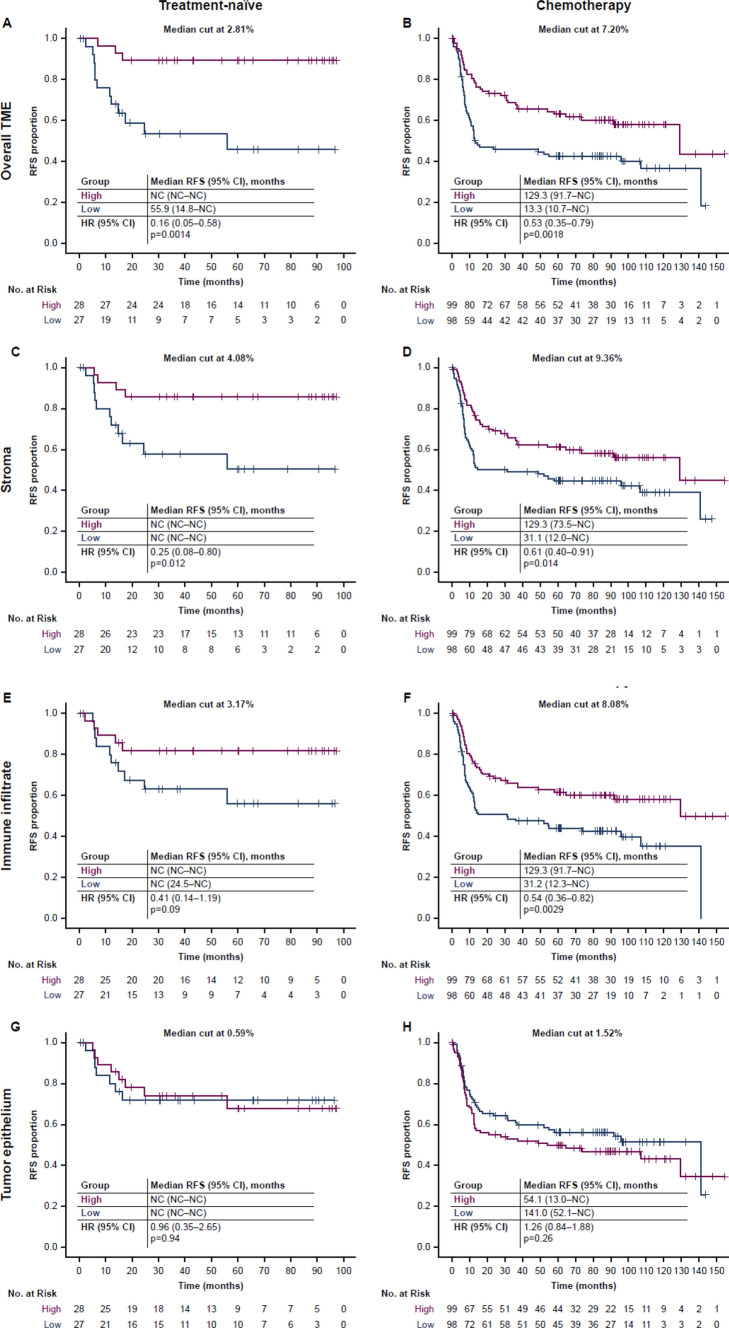
RFS in patients with ESCC who received no treatment or chemotherapy prior to surgery according to high versus low CD73 expression (dichotomized by the respective medians) in **A**, **B** the overall TME, or the **C**, **D** stromal, **E, F** immune infiltrate, or **G**, **H** epithelial compartments. In the treatment-naïve cohort, RFS was significantly longer in patients with high versus low CD73 expression in the **A** overall TME and **C** stromal compartment but not the **E** immune infiltrate or **G** epithelial compartments. In the chemotherapy cohort, RFS was significantly longer in patients with high versus low CD73 expression in the **B** overall TME, and **D** stromal and **F** immune infiltrate compartments but not the **H** epithelial compartment. Red lines represent the groups with high CD73 expression above the median cut; blue lines represent the groups with low CD73 expression below the median cut. *CD* cluster of differentiation, *CI* confidence interval, *ESCC* esophageal squamous cell carcinoma, *HR* hazard ratio, *NC* not calculable, *RFS* recurrence-free survival, *TME* tumor microenvironment

RFS was significantly longer in the CD73-high versus CD73-low groups, defined by overall TME CD73 expression, in both the treatment-naïve (median RFS NR vs 55.9 months, HR 0.16, 95% CI 0.05–0.58, p = 0.0014) (Fig. 4A) and chemotherapy (median RFS 129.3 vs 13.3 months, HR 0.53, 95% CI 0.35–0.79, p = 0.0018) (Fig. 4B) cohorts. Assessment of RFS by CD73 expression in the epithelial, stromal, and immune compartments demonstrated differences in the contributions by compartment to the RFS benefit. Analysis of RFS by CD73 expression in the stromal compartment, in which CD73 expression was higher than in the other compartments (Fig. 2), showed that there was a pronounced RFS benefit in the CD73-high versus CD73-low group in both the treatment-naïve (median RFS NR vs NR, HR 0.25, 95% CI 0.08–0.80, p = 0.012) (Fig. 4C) and chemotherapy (median RFS 129.3 vs 31.1 months, HR 0.61, 95% CI 0.40–0.91, p = 0.014) (Fig. 4D) cohorts. Similarly, benefit was seen in the CD73-high versus CD73-low group on analysis of RFS by CD73 expression in the immune infiltrate compartment in both the treatment-naïve (median RFS NR vs NR, HR 0.41, 95% CI 0.14–1.19, p = 0.09) (Fig. 4E) and chemotherapy (median 129.3 vs 31.2 months, HR 0.54, 95% CI 0.36–0.82, p = 0.0029) (Fig. 4F) cohorts. In contrast, analysis of RFS by CD73 expression in the epithelial compartment, in which CD73 expression was limited (Fig. 2), demonstrated no difference in RFS between the CD73-high and CD73-low groups in the treatment-naïve cohort (median NR vs NR, HR 0.96, 95% CI 0.35–2.65, p = 0.94) (Fig. 4G) and a numerically shorter RFS in the CD73-high versus CD73-low group in the chemotherapy cohort (median 54.1 vs 141.0 months, HR 1.26, 95% CI 0.84–1.88, p = 0.26) (Fig. 4H). Interestingly, the group with high tumor epithelial CD73 expression had lower CD8-positive and FoxP3-positive cell densities compared with the corresponding CD73-low group, whereas there were no significant differences in these cell densities between the CD73-high and CD73-low groups defined by stromal CD73 expression (Supplementary Table 1).

## Discussion

As treatment with immune-checkpoint inhibitors has become the standard of care for advanced ESCC [23], greater understanding of the TME is needed to enhance patient benefit through patient selection or identifying rational combination strategies [24].

Our findings demonstrate that neoadjuvant chemotherapy and CRT increased CD73 expression across all compartments in the TME, with the highest expression levels seen in the stromal compartment. Additionally, these data show that total CD73 expression in the TME is a positive prognostic marker, and that this is primarily driven by stromal CD73 expression. CD73 expression has already been shown to be a positive prognostic marker in breast [25], gastric [26, 27], and rectal adenocarcinoma [28]. A previous study from Taiwan in treatment naïve patients with ESCC delivered different results, where high CD73 expression was associated with worse DFS and OS [11]. However, this study did not assess stromal CD73 expression, as IHC scoring on CD73 was based on an immune reactive score (IRS) [29], which focuses on tumor staining. Furthermore, high CD73 expression was defined as a sample with a semiquantitative sum score of > 6, which was seen in 64/167 (38.3%) patients [11], in contrast to the definition in our analysis based on the median value.

A number of studies of oleclumab have evaluated CD73 as a biomarker in different treatment settings, including the first-in-human study in patients with advanced solid tumors [30], the phase Ib/II BEGONIA study in locally advanced/metastatic triple-negative breast cancer [31], which included an arm evaluating durvalumab plus oleclumab, and the phase I/randomized phase II SYNERGY study of durvalumab plus oleclumab in locally advanced/metastatic triple-negative breast cancer [32]. These studies all showed that tumor cell CD73 expression was insufficient as a biomarker. This is particularly true in the context of studies using archival samples, as exemplified by the phase II COAST study [33], because radiation and chemotherapy upregulate CD73 and often there are no subsequent tumor biopsy samples. Changes in CD73 expression during the course of treatment, as demonstrated by the elevated CD73 expression seen in the chemotherapy and CRT cohorts in the present study, indicate that they perhaps appear transiently, depending on the treatment received.

This study has several limitations. As it was a retrospective assessment of patients with ESCC, there were differences in patient characteristics between cohorts. The treatment-naïve cohort of patients who received surgery alone included a numerically higher percentage with earlier-stage disease than both the chemotherapy and CRT cohorts, which could be a confounding factor when comparing the effects of the adenosine pathway across cohorts. However, using a multivariable regression analysis with disease stage as the confounding factor, the percentage of CD73-positive tissue in the TME remained a prognostic biomarker for both RFS and OS in the chemotherapy cohort (Supplementary Fig. 5). Additionally, CD73 was scored using an AI-based methodology that differs from the IRS [29] or percent tumor or stromal cell positivity [30, 33, 34] as a scoring methodology. Larger, controlled, prospective studies are needed to build on and validate these findings.

In conclusion, our study suggested that CD73 expression is an important prognostic factor in patients with ESCC, with higher CD73 expression seen after treatment with neoadjuvant chemotherapy or CRT. Therefore, CD73 may be an important immune target for improving treatment of ESCC. Overall, this study provides the rationale for potential combination strategies of chemotherapy or CRT plus CD73-targeted therapies in ESCC.

## Supplementary Information

Supplementary Material 1

## Data Availability

Data underlying the findings described in this manuscript may be obtained in accordance with AstraZeneca’s data sharing policy described at https://astrazenecagrouptrials.pharmacm.com/ST/Submission/Disclosure. Data for studies directly listed on Vivli can be requested through Vivli at www.vivli.org. Data for studies not listed on Vivli could be requested through Vivli at https://vivli.org/members/enquiries-about-studies-not-listed-on-the-vivli-platform/. AstraZeneca’s Vivli member page is also available outlining further details: https://vivli.org/ourmember/astrazeneca/.
