# Supplementary material for: Quantifying CD73 expression after chemotherapy or chemoradiotherapy in esophageal squamous cell carcinoma
Source: Discov Oncol. 2025 Mar 29;16:427. doi: 10.1007/s12672-025-02179-x (PMC11954769; doi:10.1007/s12672-025-02179-x)
Supplement: Supplementary file 1 — Supplementary Material 1 [file 12672_2025_2179_MOESM1_ESM.docx]

**Supplementary Table 1** Immune biomarker levels according to stromal and tumor epithelial CD73 expression levels in resected tumor samples (*N* = 275)

| **Immune markers, mean (±SD)** | **Epithelial CD73 expression** | | | **Stromal CD73 expression** | | |
| --- | --- | --- | --- | --- | --- | --- |
|  | **Low^a^** | **High^a^** | ***p*-value** | **Low^a^** | **High^a^** | ***p*-value** |
| **% PD-L1-positive tissue** | 3.65 (±11.05) | 3.37 (±8.01) | 0.812 | 2.78 (±8.46) | 4.24 (±10.64) | 0.213 |
| **CD8-positive cells/mm^2^** | 296.06 (±378.76) | 177.18 (±247.01) | 0.003 | 210.97  (±313.24) | 260.36 (±334.24) | 0.222 |
| **FoxP3-positive cells/mm^2^** | 556.24 (±839.69) | 214.89 (±263.34) | < 0.0001 | 403.29 (±708.97) | 373.91 (±586.47) | 0.720 |
| **% CD39-positive tissue** | 5.46 (±6.06) | 5.28 (±4.27) | 0.795 | 4.05 (±2.99) | 6.29 (±6.08) | 0.0004 |

^a^Low/high cut-off value based on median CD73 level. Median epithelial CD73 expression = 1.3%. Median stromal CD73 expression = 8.6%.

*CD* cluster of differentiation, *PD-L1* programmed death-ligand 1, *SD* standard deviation

**Supplementary Fig. 1** Representative image of HALO^®^ artificial intelligence digital image analysis for the classification of different compartments in resected ESCC tumor samples


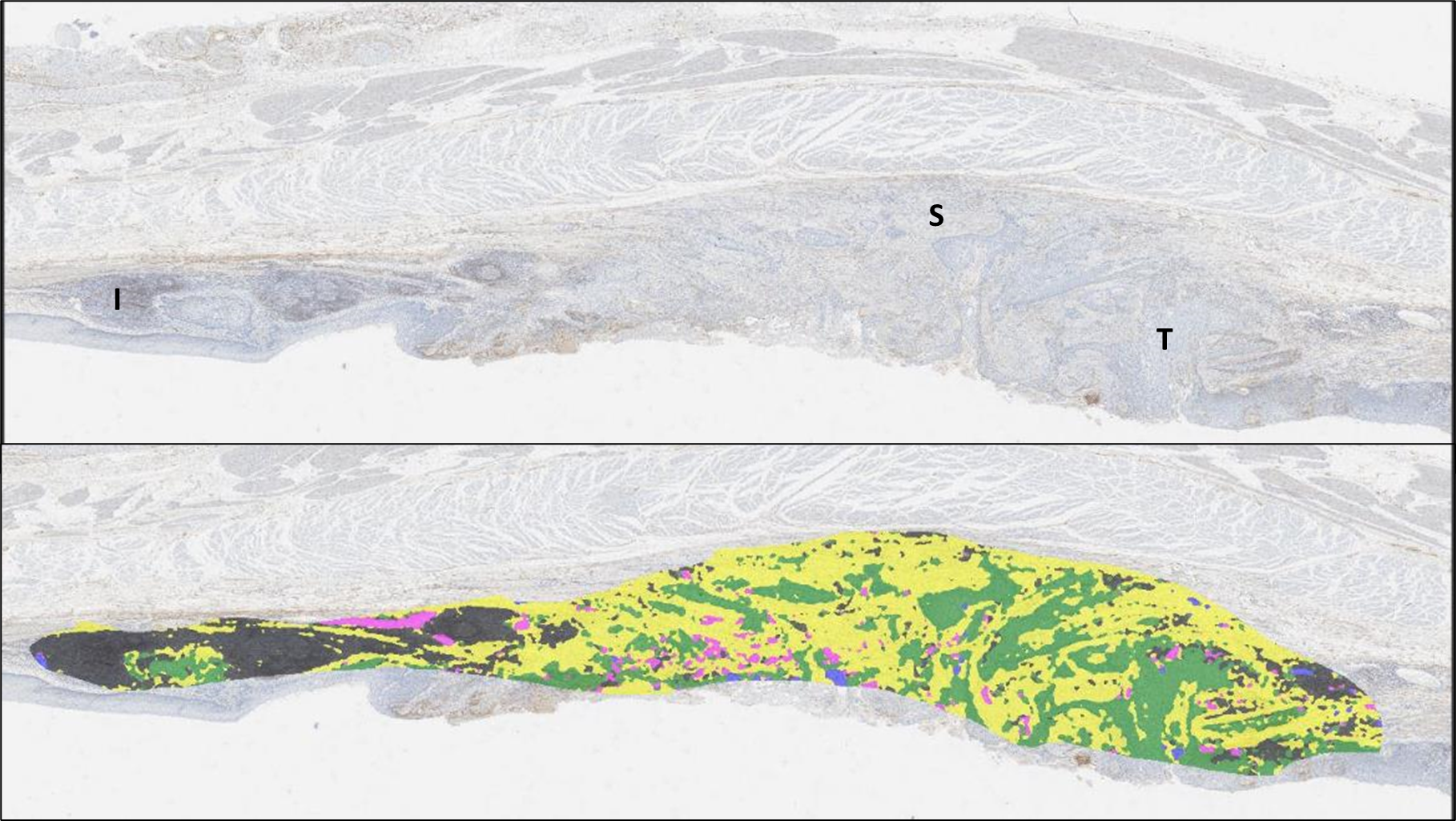


I (grey), immune infiltrates; S (yellow), stroma; T (green), tumor epithelium; and necrotic areas (purple).

*ESCC* esophageal squamous cell carcinoma

**Supplementary Fig. 2** Expression of (**a**) CD8 and (**b**) FoxP3 in tumor epithelium or stromal compartments of resected ESCC tumor samples from patients who received no treatment prior to surgery. (**a**) CD8-positive cell density was higher in the stroma versus the tumor epithelium, whereas (**b**) FoxP3-positive cell densities were similar between compartments.

Solid red lines represent the medians; dashed red lines represent the quartiles. *****p* < 0.0001.


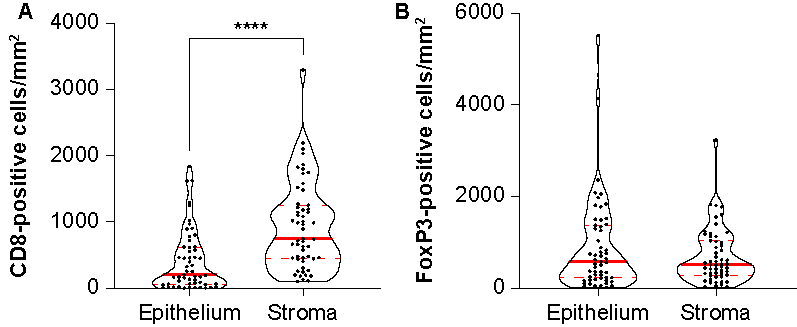


*CD* cluster of differentiation, *ESCC* esophageal squamous cell carcinoma

**Supplementary Fig. 3** (**a, b, c**) RFS and (**d, e, f**) OS in patients with ESCC who received (**a, d**) no treatment, (**b, e**) chemotherapy, or (**c, f**) CRT prior to surgery. Median RFS was (**a**) not reached, (**b**) 95.9 months, and (**c**) 6.3 months, and median OS was (**d**) 99.6, (**e**) 141.2, and (**f**) 25.6 months in the treatment-naïve, chemotherapy, and CRT cohorts, respectively.


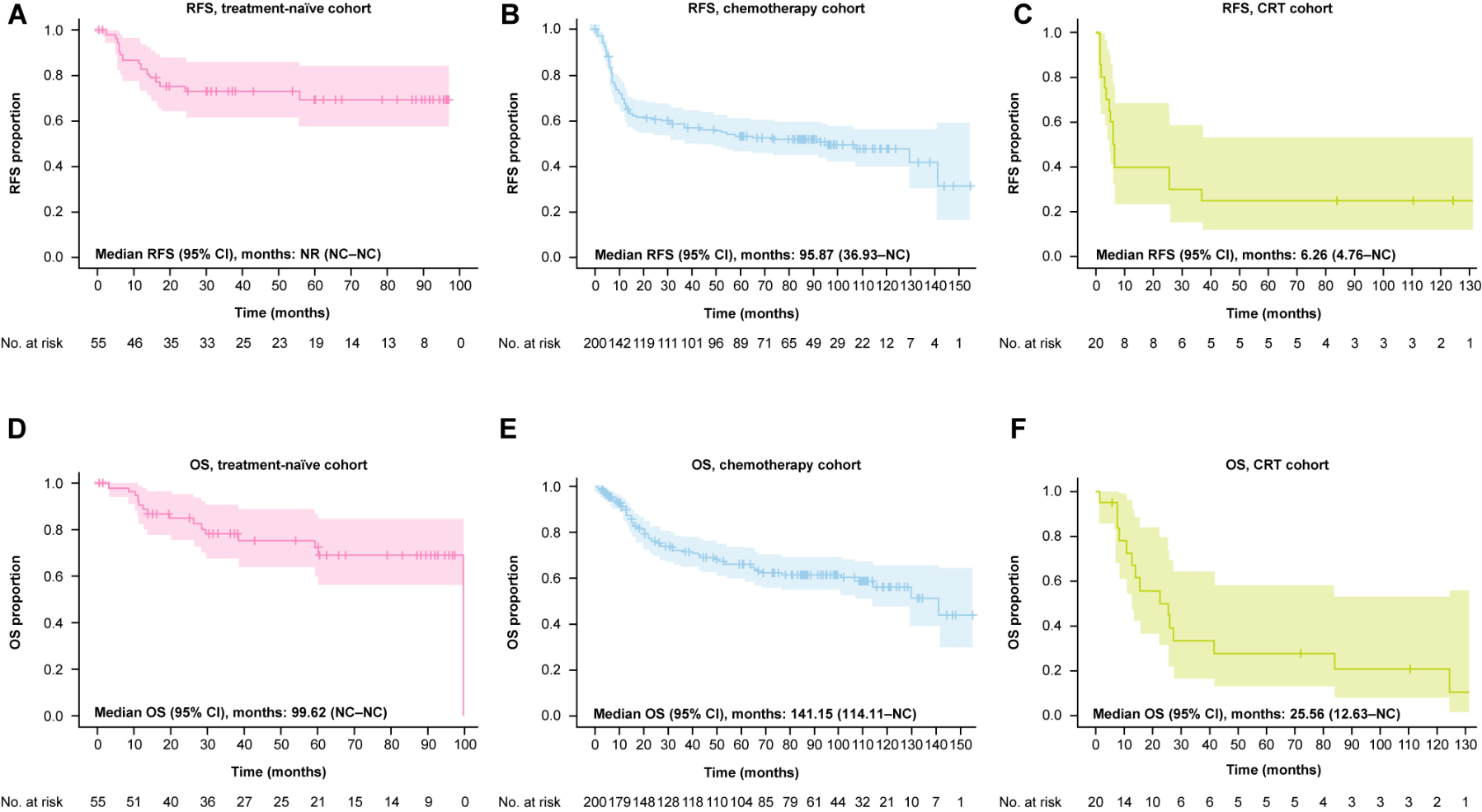


*CI* confidence interval, *CRT* chemoradiotherapy, *ESCC* esophageal squamous cell carcinoma, *NC* not calculable, *NR* not reached, *OS* overall survival, *RFS* recurrence-free survival

**Supplementary Fig. 4** Exploratory RFS and OS analyses by low versus high biomarker levels (dichotomized around the respective medians) in patients with ESCC who received no treatment or chemotherapy prior to surgery. The percentage tumor epithelium in a sample was a negative biomarker and the percentage of CD73-positive tissue in the TME was a prognostic biomarker for both RFS and OS in the treatment-naïve and chemotherapy cohorts.


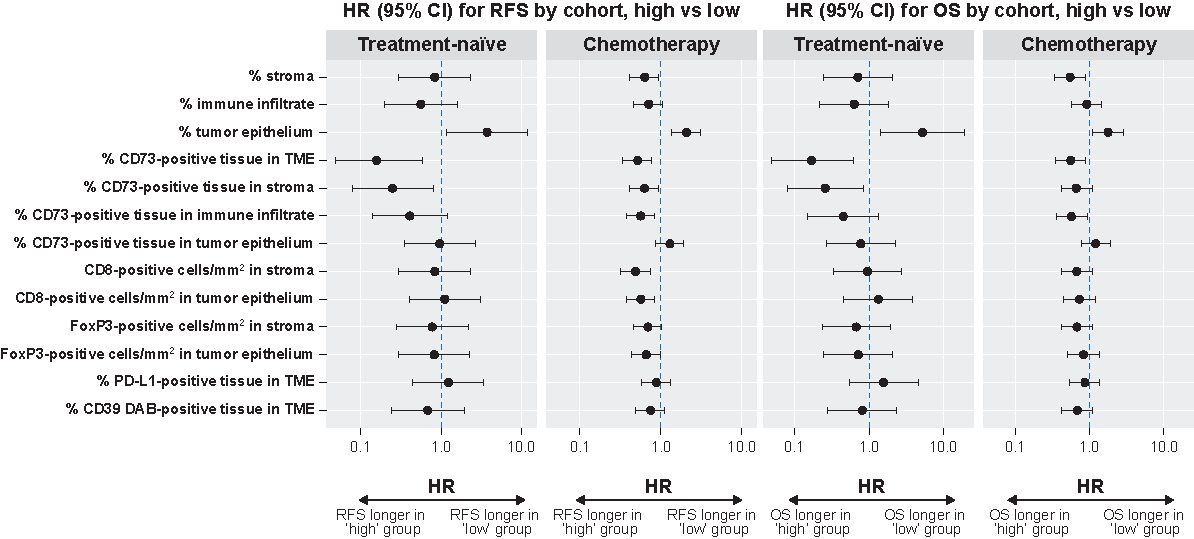


HR calculations were performed using univariant Cox regression models.

*CD* cluster of differentiation, *CI* confidence interval, *ESCC* esophageal squamous cell carcinoma, *HR* hazard ratio, *PD-L1* programmed death-ligand 1, *RFS* recurrence-free survival, *TME* tumor microenvironment, *OS* overall survival

**Supplementary Fig. 5** Exploratory multivariable RFS and OS analyses by low versus high biomarker levels (dichotomized around the respective medians) in patients with ESCC who received no treatment or chemotherapy prior to surgery, with disease stage as a confounder. The percentage tumor epithelium in a sample remains a negative biomarker and the percentage of CD73-positive tissue in the TME was a prognostic biomarker for both RFS and OS in the chemotherapy cohort.


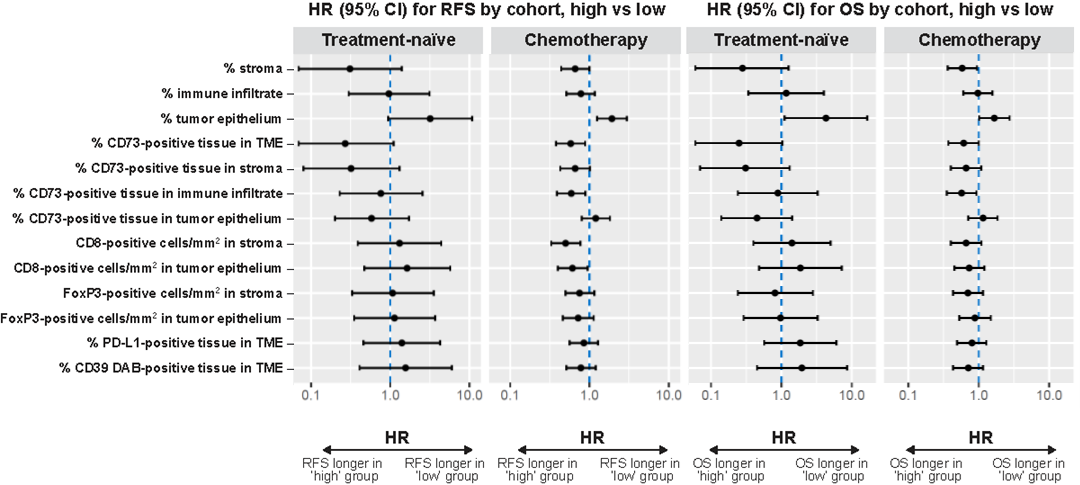


HR calculations were performed using multivariate Cox regression models where stage alone was added as a covariate.

*CD* cluster of differentiation, *CI* confidence interval, *ESCC* esophageal squamous cell carcinoma, *HR* hazard ratio, *PD-L1* programmed death-ligand 1, *RFS* recurrence-free survival, *TME* tumor microenvironment, *OS* overall survival
